# Blood lead levels and lung cancer mortality: An updated analysis of NHANES II and III

**DOI:** 10.1002/cam4.3943

**Published:** 2021-05-07

**Authors:** Jongeun Rhee, Barry I. Graubard, Mark P. Purdue

**Affiliations:** ^1^ Occupational and Environmental Epidemiology Branch Division of Cancer Epidemiology and Genetics National Cancer Institute Rockville Maryland USA; ^2^ Biostatistics Branch Division of Cancer Epidemiology and Genetics National Cancer Institute Rockville Maryland USA

**Keywords:** blood lead, lead, lung cancer, lung cancer mortality, NHANES

## Abstract

Previous analyses within the National Health and Nutrition Examination Survey (NHANES) II and III cycles suggested an association between blood lead levels (BLLs) and lung cancer mortality, although the evidence was limited by small case numbers. To clarify this relationship, we conducted updated analyses of 4,182 and 15,629 participants in NHANES II and III, respectively, (extending follow‐up 20 and 8 years) aged ≥20 with BLL measurements and mortality follow‐up through 2014. We fit multivariable Cox models to estimate hazard ratios (HRs) and 95% confidence intervals (CIs) relating BLLs and lung cancer with adjustment for smoking and other factors. We did not observe an overall association between BLLs and lung cancer after adjustment for smoking (both surveys) and serum cotinine and environmental tobacco smoke exposure (NHANES III), although suggestive associations were observed among women (NHANES II: HR 2.7, 95% CI 0.7, 10.0 for ≥20.0 µg/dl vs. <10.0 µg/dl, *P*_trend_ = 0.07; NHANES III: HR 11.2, 95% CI 2.1, 59.4 for ≥10.0 µg/dl vs. <2.5 µg/dl, *P*_trend_ = 0.04). After stratifying on smoking status, an association with elevated BLLs was observed in NHANES II only among former smokers (HR 3.2, 95% CI 1.3, 8.0 for ≥15 vs. <15 µg/dl) and in NHANES III only among current smokers (HR 1.7, 95% CI 1.1, 2.8 for ≥5 vs. <5 µg/dl).

In summary, we found elevated BLLs to be associated with lung cancer mortality among women in both NHANES II and III. Given the absence of an association among non‐smokers, we cannot rule out residual confounding as an explanation for our findings.

## Graphical abstract

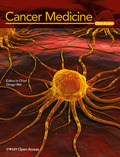

**Abbreviations**
BLL: blood lead level
CI: confidence interval
ETS: environmental tobacco smoke
HR: hazard ratio
IARC: International Agency for Research on Cancer
NCHS: National Center for Health Statistics
NDI: National Death Index
NHANES: National Health and Nutrition Examination Survey
RR: relative risk

## 1 INTRODUCTION

Environmental exposure to lead in the United States has substantially decreased due to the elimination of leaded gasoline since the 1970’s.^1^ However, high lead exposures persist in several occupations, such as lead–acid battery workers and miners, and is a major public health threat in communities with lead‐contaminated drinking water due to corrosion of plumbing materials.^2^, ^3^, ^4^ Lead exposure is known to be harmful, particularly for children; established health effects include damage to the brain and nervous system, gastrointestinal problems, anemia, liver and kidney damage, infertility, and developmental delays.^5^ Lead is also a suspected carcinogen. In 2004, a Working Group for the International Agency for Research on Cancer (IARC) Monographs on the Evaluation of Carcinogenic Risks to Humans classified inorganic lead as a probable carcinogen (Group 2A) based on sufficient evidence in experimental animals and limited evidence in humans.^2^ According to the Working Group, the most informative general population studies at the time were two analyses of blood lead levels (BLLs) and cancer mortality among 3,592 and 4,292 adult participants in the second National Health and Nutrition Examination Survey (NHANES II, conducted between 1976 and 1980) followed up through 1992.^6^, ^7^ While both analyses reported suggestive evidence of an exposure–response relationship between elevated BLLs and lung cancer, the Working Group noted that the level of control for smoking history in each analysis was comparatively crude, raising the potential for residual confounding from smoking to have partly accounted for the apparent associations. A later mortality analysis of BLLs and cancer mortality among 3,482 adult participants in the third NHANES (NHANES III, 1988–1994) followed through 2006 also reported an association with lung cancer, although the adjustment for smoking was again limited.^8^ None of the analyses reported findings stratifying on smoking status, probably due to an inadequate number of cases.

In 2020, inorganic lead was recommended by an IARC Advisory Group as a high priority for evaluation by the IARC Monographs Program.^9^ To clarify the relationship between BLLs and lung cancer mortality and help to inform a reevaluation of lead, we conducted an updated analysis within NHANES II and III with mortality linkage extended through December 2014.

## 2 MATERIALS AND METHODS

### 2.1 Study population

NHANES II (1976–1980) and NHANES III (1988–1994) used a stratified multistage probability survey design to select a representative sample of the civilian noninstitutionalized US population (https://wwwn.cdc.gov/nchs/nhanes/).^10^, ^11^ Both surveys included a household interview collecting information on medical history and demographic and socioeconomic characteristics, and a standardized physical exam in a mobile examination center. There were 12,520 and 17,330 adults who were age 18 or older at the time of examination in NHANES II and NHANES III, respectively.

### 2.2 Exposure measurement and outcome ascertainment

During the physical examination, whole‐blood specimens were collected by venipuncture for all survey participants ≥6 months of age in NHANES II ^11^ and ≥1 year of age in NHANES III.^12^ Blood lead concentrations (µg/dl) were measured using an atomic absorption spectrophotometer in NHANES II ^13^ and a graphite furnace atomic absorption spectrophotometer in NHANES III.^14^

NHANES II and NHANES III participants ≥18 years of age and with sufficient identifying data were followed for mortality status through December 2014 by probabilistic linkage with the National Death Index (NDI).^15^ The International Classification of Diseases, Ninth Revision (ICD‐9) (in NHANES II) and Tenth Revision (ICD‐10) (in NHANES III) were used to identify deaths due to malignant neoplasms of the trachea, bronchus, and lung (ICD‐9 162; ICD‐10 C34).^16^, ^17^

**FIGURE 1:**
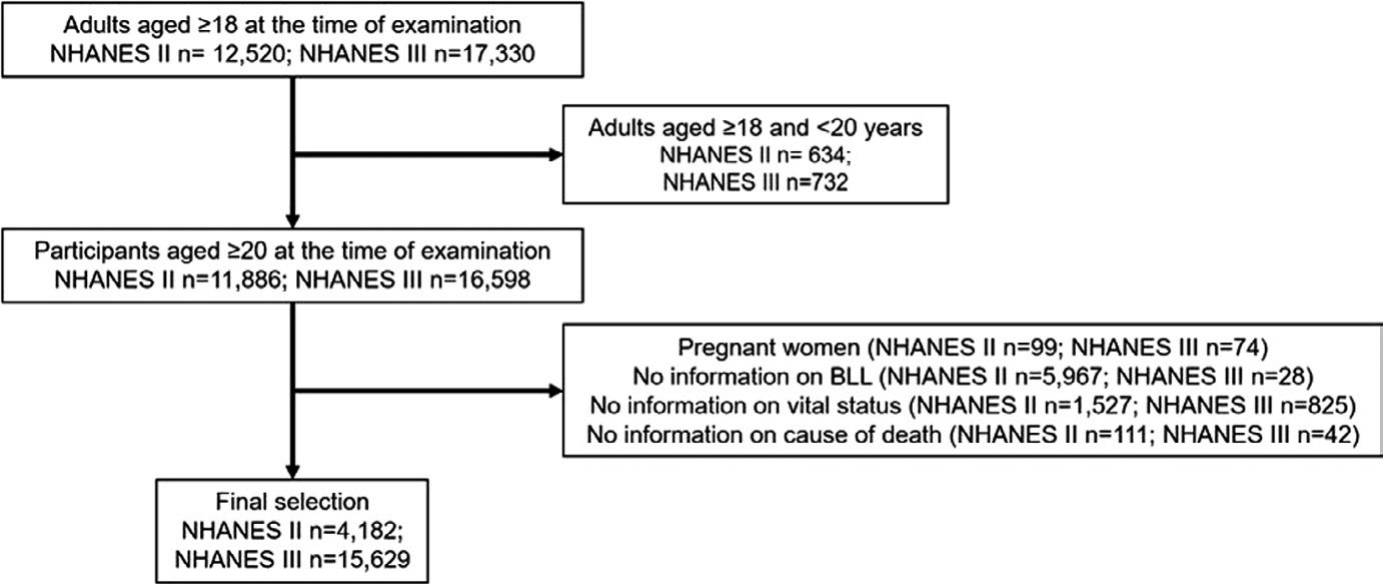
Flow chart of participant selection in the analytic cohort within NHANES II and III. Abbreviations: NHANES, National Health and Nutrition Examination Survey, BLL: blood lead level

For this investigation we limited our study population to participants aged 20 years or older at examination (n = 11,886 and 16,598 for NHANES II and III, respectively). We excluded participants who were pregnant (n = 99 and 74) and without information on BLL, due to BLL only being assessed for a random half‐sample of NHANES II participants or with missing values (n = 5,967 and n = 28). We also excluded participants with no information on vital status (n = 1,527, n = 825) or cause of death (n = 111, n = 42). The analytic data set included 4,182 participants and 15,629 participants in NHANES II and NHANES III, respectively (Figure 1).

This project was approved by the National Center for Health Statistics (NCHS) Review Committee. The statistical analyses were conducted at a Research Data Center given the use of restricted data from the mortality linkage. The project was classified as not human subjects research given the lack of investigator access to personally identifiable information.

### 2.3 Statistical analyses

We summarized selected participant demographic and lifestyle characteristics across study‐specific categories of BLL (<10.0, 10.0–14.9, 15.0–19.9, and ≥20.0 µg/dl in NHANES II and <2.5, 2.5–4.9, 5.0–9.9, and ≥10.0 µg/dl in NHANES III). We fit multivariable Cox proportional hazard models to estimate hazard ratios (HRs) and 95% confidence intervals (CIs) for BLL with death from lung cancer using attained age as the underlying time metric and adjusting for sex, race/ethnicity (non‐Hispanic white, non‐Hispanic black, Mexican American, and other), education level (<12 years, ≥12 years of education), urban/rural index, poverty income ratio, body mass index (<25, ≥25 and <30, and ≥30 kg/m^2^), and smoking (never, former, current, and <median pack‐years (27 years for NHANES II and 13.3 years for NHANES III), current and ≥median pack‐years). In NHANES III we also ran multivariable models additionally adjusting for serum cotinine (per ng/mL increase), number of household residents who smoke cigarettes at home (0, 1, and ≥2), and number of working hours per day exposed to tobacco smoke (0, 1–4, and >4) to control for environmental tobacco smoke (ETS) exposure. We modeled BLL as a categorical variable using <10.0, 10.0–14.9, 15.0–19.9, and ≥20.0 µg/dl in NHANES II and <2.5, 2.5–4.9, 5.0–9.9, and ≥10.0 µg/dl in NHANES III as cut‐points. A test of trend was performed by modeling intra‐category medians as a continuous covariate. For the Cox models the baseline hazard was stratified by examination year and 6‐year intervals of birth cohort to account for the decline in BLLs over the years.^18^ We conducted separate analyses for NHANES II and III since BLLs had declined substantially between the two survey cycles.^6^, ^8^

We also conducted stratified analyses of dichotomized categories of BLLs (NHANES II: <15 or ≥15 µg/dl; NHANES III: <5 and ≥5 µg/dl) by smoking status (never, former, and current). We observed no indication of a violation of the proportional hazards assumption after testing for interaction between continuous BLL and attained age.

Statistical analyses were conducted using the SAS System for Windows (release 9.1; SAS Institute Inc., Cary, NC) and SUDAAN (release 11.0; Research Triangle Institute, Research Triangle Park, NC). All analyses included sample weights that accounted for the unequal probabilities of selection and nonresponse and the complex sample design. All statistical tests were two‐sided, and *P* values less than 0.05 were considered statistically significant.

## 3 RESULTS

In this extended mortality linkage, a total of 189 and 363 lung cancer deaths (out of 3,065 and 6,158 total deaths) occurred in the NHANES II and III analytic sets with 103,864 and 287,976 person‐years of follow‐up, respectively. At baseline, persons with higher BLLs were more likely to be male, non‐Hispanic black, current smokers, wealthier, to live in urban areas, and have less than 12 years of education in NHANES II (Table 1). In NHANES III, similar patterns were observed, but subjects with higher BLLs additionally tended to be older and poorer. The median BLLs in NHANES II and NHANES III were 13.0 μg/dl and 3.0 μg/dl, respectively.

**TABLE 1:** Characteristics of NHANES II and NHANES III participants^a^ by category of blood lead level

Characteristics	Blood lead level (µg/dl)									
	NHANES II					NHANES III				
	<10.0	10.0–14.9	15.0–19.9	>20.0	All	<2.5	2.5–4.9	5.0–9.9	>10.0	All
	(n = 856)	(n = 1,663)	(n = 1,051)	(n = 723)	(n = 4293)	(n = 5,659)	(n = 5,883)	(n = 3,330)	(n = 799)	(n = 15,671)
Age at baseline, mean	43.9	46.1	46.7	46	45.7	40.5	48.5	51.8	51.5	48.1
Poverty income ratio^a^, mean	2.2	2.5	2.7	2.7	2.5	2.5	2.2	1.8	1.6	2.0
Sex, %										
Male	16.5	40.6	60.6	80	49.4	32.1	56.2	70.2	81	59.9
Female	83.5	59.4	39.4	20	50.6	67.9	43.8	29.8	19	40.1
Race, %										
Non‐Hispanic white	73.3	75.3	75	72.6	74.1	78.6	77.9	72.2	64.9	73.4
Non‐Hispanic black	6.3	8.7	10.8	13.4	9.8	8.9	10.1	13.6	20.3	13.2
Mexican American	3	2.4	1.4	2.3	2.3	4.7	4.8	5.8	6.7	5.5
Other	17.4	13.6	12.7	11.8	13.9	7.8	7.2	8.4	8	7.9
Smoking status, %										
Never	57.7	41.5	33.6	18.5	37.8	57.4	40.9	28.4	20.4	36.8
Former	19.5	23.9	25.9	25.9	23.8	23	27.7	30.6	27	27.1
Current	22.8	34.6	40.4	55.5	38.3	19.6	31.4	41	52.6	36.2
Body mass index (kg/m^2^), %										
<25	56.1	51.9	47.4	45.9	50.3	47.4	41.7	43.6	47.2	45.0
25–30	26.9	33.4	36.7	38.9	34.0	28.8	35.8	37.2	36.1	34.5
≥30	17.1	14.7	15.9	15.3	15.8	23.8	22.5	19.2	16.6	20.5
Urban status, %	56.8	68	70.7	73.9	67.4	47.2	47.9	47.7	49.3	48.0
Less than 12 years of education, %	33	31.4	34.4	39.6	34.6	17.4	26.8	37.6	43.8	31.4

Note

*P* for group differences were significant (*p* <0.05) for all covariates in both surveys except BMI in NHANES II (*p*=0.07).

Abbreviations: NHANES, National Health and Nutrition Examination Survey.

^a^ Included study subjects were participants age 20 or older and non‐pregnant at medical examination with personal information (age at baseline, poverty income ration, sex, race, smoking status, body mass index, urban status, and education), information on blood lead level, and vital status. Analytic cohort includes 4,182 and 15,629 participants in NHANES II and III, respectively, after excluding subjects without cause of death (n=111 and n=42).

† Poverty income ratios of less than 1.0 can be described as “below poverty” and ratios greater than or equal to 1.0, as “at or above poverty.”

We summarized in Table 2 our findings relating categories of BLL to lung cancer mortality. In multivariable models excluding smoking, we observed statistically significant associations between elevated BLLs and lung cancer mortality in NHANES II (HR 3.1, 95% CI 1.1, 8.7 for BLL ≥20.0 µg/dl vs. <10.0 µg/dl, *P*_trend_ = 0.006) and NHANES III (HR 3.3, 95% CI 1.6, 6.7 for BLL ≥10.0 µg/dl vs. <2.5 µg/dl, *P*_trend_ = 0.00009). After additionally adjusting for smoking, however, these associations weakened substantially and were no longer statistically significant (NHANES II: HR 1.8, 95% CI 0.7, 4.7 for BLL ≥20.0 µg/dl, *P*_trend_ = 0.12; NHANES III: HR 1.4, 95% CI 0.9, 2.0 for BLL ≥10.0 µg/dl, *P*_trend_ = 0.08). The association in NHANES III became null with additional adjustment for serum cotinine and ETS exposure (HR 1.2, 95% CI 0.5, 3.4 for BLL ≥10.0 µg/dl, *P*_trend_ = 0.21).

**TABLE 2:** Hazard ratios relating categories of blood lead levels and lung cancer mortality with varying adjustment for tobacco smoke exposure among NHANES II and NHANES III participants age 20 or older and non‐pregnant at medical examination; follow‐up through 31 December 2014

Survey	Blood Lead Level (µg/dl)	N_Deaths_	Base Model^a^	Base Model + Smoking^b^	Base Model +Smoking, Serum Cotinine, Environmental Tobacco Smoke Exposure^c^
			HR (95% CI)	HR (95% CI)	HR (95% CI)
NHANES II	<10.0	17	1	1	
	10.0–14.9	65	1.4 (0.7, 2.9)	1.1 (0.5, 2.4)	
	15.0–19.9	55	2.0 (0.8, 5.0)	1.4 (0.6, 3.4)	
	≥20.0	52	**3.1 (1.1, 8.7)**	1.8 (0.7, 4.7)	
			***P*_trend_ = 0.006**	*P*_trend_ = 0.12	
NHANES III	<2.5	77	1	1	1
	2.5–4.9	107	0.9 (0.6, 1.3)	0.6 (0.4, 0.9)	0.5 (0.3, 0.9)
	5.0–9.9	126	**1.8 (1.1, 2.9)**	0.9 (0.5, 1.6)	1.0 (0.5, 2.1)
	≥10.0	53	**3.3 (1.6, 6.7)**	1.4 (0.9, 2.0)	1.2 (0.5, 3.4)
			***P*_trend_ = 0.00009**	*P*_trend_ = 0.08	*P*trend = 0.21

Note

*P* for trend estimated from modeling the intra‐category medians.

Abbreviations: CI, confidence interval; HR, hazard ratio; NHANES, National Health and Nutrition Examination Survey.

^a^ Hazard ratios were computed from Cox proportional hazards models using age as the time scale, baseline hazard was stratified by birth year (6‐year categories) and examination year. Models were adjusted for sex, race/ethnicity, education level, urban/rural index, poverty income ratio, and body mass index.

^b^ Hazard ratios were computed as above with additional adjustment for smoking (never, former, current and <median pack‐years, and current and ≥median pack‐years). Median pack‐years: 27 years in NHANES II and 13.3 years in NHANES III.

^c^ Hazard ratios were computed as above with additional adjustment for serum cotinine (per ng/mL increase), number of household residents who smoke cigarettes in home (0, 1, and ≥2,) and number of working hours per day exposed to tobacco smoke (0, 1–4, and >4). Information on serum cotinine and environmental tobacco smoke exposure was only available in NHANES III.

When we conducted smoking‐adjusted analyses stratified by sex (Table 3), we observed stronger associations for lung cancer mortality among women in each study population (NHANES II: HR 2.7, 95% CI 0.7, 10.0 for BLL ≥20.0 µg/dl, *P*_trend_ = 0.07; NHANES III: HR 2.7, 95% CI 1.2, 6.5 for BLL ≥10.0 µg/dl, *P*_trend_ = 0.03), while null findings were observed among men. When we further adjusted for cotinine and ETS exposure in NHANES III, the association among women remained (HR 11.2, 95% CI 2.1, 59.4 for BLL ≥10.0 µg/dl, *P*_trend_ = 0.04).

**TABLE 3:** Hazard ratios relating categories of blood lead levels and lung cancer mortality separately among male and female NHANES II and NHANES III participants age 20 or older and non‐pregnant at medical examination; follow‐up through December 31, 2014

Survey	Blood Lead Level (µg/dl)	Men			Women		
		N_Deaths_	Base Model + Smoking^a^	Base Model +Smoking, Serum Cotinine, Environmental Tobacco Smoke Exposure^b^	N_Deaths_	Base Model + Smoking^a^	Base Model +Smoking, Serum Cotinine, Environmental Tobacco Smoke Exposure^b^
			HR (95% CI)	HR (95% CI)		HR (95% CI)	HR (95% CI)
NHANES II^c^	<10.0		1			1	
	10.0–14.9		0.7 (0.3, 1.8)			1.5 (0.6, 3.9)	
	15.0–19.9		0.9 (0.4, 2.4)			2.0 (0.7, 6.1)	
	≥20.0		1.1 (0.4, 2.9)			2.7 (0.7, 10.0)	
			*P*_trend_ =0.28			*P*_trend_ =0.07	
NHANES III	<2.5	28	1	1	49	1	1
	2.5–4.9	68	0.6 (0.3, 1.1)	0.5 (0.2, 1.0)	39	0.5 (0.3, 1.0)	0.5 (0.2, 1.6)
	5.0–9.9	88	0.8 (0.4, 1.6)	0.7 (0.3, 1.5)	38	1.3 (0.6, 2.8)	1.9 (0.7, 5.1)
	≥10.0	41	1.1 (0.5, 2.5)	0.8 (0.3, 2.0)	12	**2.7 (1.2, 6.5)**	**11.2 (2.1, 59.4)**
			*P*_trend_ =0.31	*P*_trend_ =0.69		***P*_trend_ =0.03**	***P*_trend_ =0.04**

Note

*P* for trend estimated from modeling the intra‐category medians.

Bold values are considered statistically significant.

Abbreviations: CI, confidence interval; HR, hazard ratio; NHANES, National Health and Nutrition Examination Survey.

^a^ Hazard ratios were computed from Cox proportional hazards models using age as the time scale, baseline hazard was stratified by birth year (6‐year categories) and examination year. Models were adjusted for sex, race/ethnicity, education level, urban/rural index, poverty income ratio, body mass index, and smoking (never, former, current and <median pack‐years, and current and ≥median pack‐years). Median pack‐years: 27 years in NHANES II and 13.3 years in NHANES III.

^b^ Hazard ratios were computed as above with additional adjustment for serum cotinine (per ng/mL increase), number of household residents who smoke cigarettes in home (0, 1, and ≥2), and number of working hours per day exposed to tobacco smoke (0, 1–4, and >4). Information on serum cotinine and environmental tobacco smoke exposure was only available in NHANES III.

^c^ The number of deaths from lung cancer in NHANES II was suppressed by the National Center for Health Statistics (NCHS) Research Data Center.

Table 4 summarizes the analyses of dichotomized BLLs and lung cancer mortality stratifying on smoking status. An association with elevated blood lead was observed in NHANES II only among former smokers (HR 3.2, 95% CI 1.3, 8.0 for BLL ≥15 vs. <15 µg/dl) and in NHANES III only among current smokers (HR 1.7, 95% CI 1.1, 2.8 for BLL ≥5 vs. <5 µg/dl). There was no suggestion of an association between elevated blood lead and lung cancer mortality among non‐smokers, with both HRs <1.

**TABLE 4:** Hazard ratios relating blood lead levels and lung cancer mortality among NHANES II and NHANES III participants stratifying on smoking status

Survey	Smoking status	Blood lead (µg/dl)	N_Deaths_	HR (95% CI)
NHANES II	Never smokers	<15	14	1
		≥15	5	0.4 (0.1, 1.7)
	Former smokers	<15	14	1
		≥15	22	3.2 (1.3, 8.0)
	Current smokers	<15	54	1
		≥15	80	1.3 (0.8, 2.1)
NHANES III	Never smokers	<5	33	1
		≥5	3	0.1 (0.02, 0.3)
	Former smokers	<5	73	1
		≥5	56	1.3 (0.8, 2.2)
	Current smokers	<5	78	1
		≥5	120	1.7 (1.1, 2.8)

Note

Hazard ratios were computed from Cox proportional hazards models using age as the time scale, baseline hazard is stratified by birth year (6‐year categories) and examination year. Models were adjusted for sex, race/ethnicity, education level, urban/rural index, poverty income ratio, and body mass index.

Abbreviations: CI, confidence interval; HR, hazard ratio; NHANES, National Health and Nutrition Examination Survey.

## 4 DISCUSSION

In this extended analysis of blood lead and lung cancer mortality in NHANES II and NHANES III, we found elevated BLLs to be associated with increased lung cancer mortality among women in both study populations after adjustment for smoking and, in NHANES III, serum cotinine, and ETS exposure. However, there was no suggestion of this relationship in analyses of never smokers in either survey.

Our finding of stronger evidence for an association between BLLs and lung cancer among women compared to men was unexpected. However, it is notable that an earlier NHANES II analysis also observed a stronger association between BLLs and lung cancer among women (relative risk (RR) 2.5, 95% CI 0.7, 8.4 for above‐ vs. below median levels, *p* = 0.14) than among men (RR 1.2, 95% CI 0.6, 2.5, *p* = 0.66).^6^ Similarly, a recent cohort analysis of BLLs among 81,067 South Korean lead‐exposed workers (54,788 male and 26,279 female) found BLLs to be significantly related to elevated lung cancer mortality among women only.^19^ The Korean study did not adjust for smoking in the analysis, although among subjects with smoking data, the proportion of smokers among female workers with BLL ≥10 μg/dl was lower (1.9%) than for those with BLL <10 μg/dl (2.9%). A recent pooled analysis of three cohort studies (USA, Finland, and United Kingdom) however, supports an association among men.^20^ In this analysis of 88,187 workers (96% male) and 1,333 lung cancer deaths, a highly significant trend between BLLs and lung cancer was observed (HR 1.78, 95% CI 1.51, 2.08 for ≥40 µg/dL vs. <20 µg/dl, *P_trend_* < 0.0001). This study did not adjust for smoking, although in a subsample of 115 US subjects with smoking data, no association with BLLs was observed.

An important potential source of bias in this analysis is residual confounding from tobacco use, a source of lead and strong risk factor for lung cancer. In our analyses, we observed an association between elevated BLLs and lung cancer mortality among women with adjustment for smoking status and pack‐years of smoking in both surveys, and adjustment for serum cotinine concentration and ETS exposure in NHANES III. However, we did not observe any evidence of such an association among never smokers; consequently, we cannot rule out residual confounding as an explanation for our findings. That said, our finding in NHANES II of an association between elevated BLLs and lung cancer among former smokers but not current smokers is surprising and different from NHANES III, where the association is restricted to current smokers. The BLL measurements in these two study populations capture different time points in the reduction of environmental lead exposures from the mandated phaseout of lead from gasoline and paint, which began in the United States in 1974.^1^ Among the NHANES II subjects enrolled between 1976 and 1980, when the US average lead content in gasoline decreased from 1.7 to 1.2 g/L, the median BLL was 13.0 μg/dl, while among NHANES III subjects, enrolled after the average gasoline lead content had dropped to 0.1 g/L, the median BLL was 3.0ug/dL.^1^ Given these differences, and the fact that BLLs reflect relatively recent exposures (the half‐life of blood lead is approximately 1 month^21^), environmental lead exposure (i.e., to leaded gasoline engine exhaust and paint) likely accounts for a larger fraction of BLLs in NHANES II than in NHANES III and, in turn, lead from tobacco smoke may be a more important determinant of BLLs in NHANES III versus NHANES II. If this is the case, we speculate that it is possible that the association between BLLs and lung cancer observed in NHANES III may be more strongly driven by tobacco smoke exposure, and more susceptible to residual confounding from smoking, than in NHANES II.

It is unclear what biologic effects might underlie a causal association between lead exposure and lung cancer, should one exist. Potential carcinogenic mechanisms include DNA damage induced by reactive oxygen species, interference with DNA synthesis and repair processes, and alteration of tumor gene expression.^2^ The interactions of lead with genotoxic and mutagenic co‐exposures, including UV radiation, oxygen radicals, and chemical mutagens can increase genotoxicity in cells.^22^, ^23^ The potential biologic basis for a difference in the carcinogenicity of lead among women versus men is also unknown.^24^ Sex‐based differences in immunotoxic responses to low levels of lead during embryonic development have been observed both in juvenile chicken and adult rats.^25^, ^26^ A cohort study of children with early life lead exposure found sex‐dependent DNA methylation differences for several genes associated with detoxification pathways such as Glutathione Peroxidase 1 (4%–5% higher promoter methylation in females) and Cytochrome P450 1A1 (5% higher methylation status around the transcription start site in females), which may play a role in carcinogenesis.^27^, ^28^ However, the biologic rationale for a sex‐specific association between BLLs and lung cancer mortality is unclear and warrants further investigation.

A strength of this investigation, as with earlier NHANES analyses, is its prospective design and generalizability to the US population.^6^, ^7^, ^8^ Additionally, with the substantially increased number of lung cancer deaths from extended mortality follow‐up, we were able to conduct analyses stratified on sex and smoking status. A limitation of our analysis is the use of single blood lead measurements, which may not accurately reflect cumulative lead exposure as a measure of long‐term lead exposure. The use of cancer deaths as a surrogate for incident cases can be susceptible to misclassification, although death certificates have been demonstrated to have high sensitivity and specificity for capturing lung cancer.^29^

## 5 CONCLUSION

In conclusion, in our extended analysis of BLLs and lung cancer mortality in NHANES II and III we found suggestive evidence of an association between elevated BLLs and lung cancer mortality among women with adjustment for measures of tobacco exposure. However, as we did not observe this association among non‐smokers, we cannot rule out residual confounding from tobacco as an explanation for these findings. In order to clarify this relationship, there is a need for studies incorporating more detailed assessments of cumulative lead exposure, such as a cumulative blood lead index from serially collected BLLs or in vivo measurements of bone lead.^30^, ^31^ Our findings also point to a need for the inclusion of women in future epidemiologic studies of lead exposure and for careful consideration of the possibility of confounding from tobacco use.

## CONFLICT OF INTEREST

None declared.

## AUTHOR CONTRIBUTIONS

Jongeun Rhee conducted statistical analyses and wrote the manuscript. Barry I Graubard provided feedback on statistical analyses and the manuscript. Mark P. Purdue designed the study, supervised the project, conducted some statistical analyses, and critically reviewed the manuscript.

## Data Availability

The public data of NHANES II and III public are available at https://wwwn.cdc.gov/nchs/nhanes/. Use and analysis of restricted data are possible at a National Center for Health Statistics (NCHS) Research Data Center with NCHS approval of a data analysis proposal.
